# Electrical Stimulation of Trigeminal Nerve at the Anterior Nasal Septum in Healthy Individuals and Patients With Olfactory Dysfunction

**DOI:** 10.1002/alr.70003

**Published:** 2025-08-01

**Authors:** Konstantinos Garefis, Susanne Weise, Pauline Hanslik, Coralie Mignot, Halina B. Stanley, Maxime Fieux, Camille Ferdenzi, Evangelia Tsakiropoulou, Clémentine Lipp, Arnaud Bertsch, Moustafa Bensafi, Iordanis Konstantinidis, Thomas Hummel

**Affiliations:** ^1^ 2nd Academic ORL Head and Neck Surgery Department Aristotle University of Thessaloniki Papageorgiou Hospital Thessaloniki Greece; ^2^ Smell & Taste Clinic Department of Otolaryngology Technische Universität Dresden Dresden Germany; ^3^ Department of Neuropop Université Claude Bernard Lyon 1, CNRS, INSERM, Centre de Recherche en Neurosciences de Lyon CRNL U1028 UMR5292 NEUROPOP Bron France; ^4^ Hospices Civils De Lyon, Hôpital Lyon Sud, Service d'ORL, D'otoneurochirurgie Et De Chirurgie cervico‐faciale, F‐69310, Pierre Bénite; Université de Lyon, Université Lyon 1 Lyon France; ^5^ École Polytechnique Fédérale de Lausanne EPFL‐STI‐IMT‐LMIS1 Lausanne Switzerland

**Keywords:** anosmic, hyposmic, nasal septum, olfactory disorders, smell, trigeminal

## Abstract

**Background:**

The intranasal trigeminal system contributes to the sense of smell. Its integrity in olfactory dysfunction (OD) may be crucial for future treatments. This study assessed trigeminal sensitivity in healthy individuals and patients with OD of different etiologies using electrical stimulation of the nasal mucosa.

**Methods:**

Eighty‐two participants were included in this multicentric study: healthy individuals (*n* = 28) and patients with OD (*n* = 54), comprising post‐viral OD (PVOD, *n* = 29), post‐traumatic OD (PTOD, *n* = 15), and chronic rhinosinusitis with nasal polyps (SND, *n* = 10). Olfactory function was assessed with the Sniffin’ Sticks test battery; trigeminal sensitivity was measured via electrical thresholds at the anterior nasal septum.

**Results:**

Age significantly affected electrical thresholds (ET), with older individuals showing higher thresholds (*p* = 0.012). In healthy participants, ET correlated positively with age (*p* = 0.02). PTOD patients exhibited higher ET compared to healthy individuals (*p* = 0.033), while PVOD and SND patients showed no significant differences. Anosmic patients had higher ET than hyposmic (*p* = 0.001) and normosmic individuals (*p* < 0.001); hyposmic and normosmic individuals did not differ significantly. Among age, etiology, and olfactory function, olfactory function was the most significant factor influencing ET (*p* = 0.013). All participants rated the stimulation as moderately intense and mildly painful, with ratings increasing with higher currents.

**Conclusions:**

Intranasal trigeminal function was largely preserved in OD patients, especially in hyposmic individuals. PTOD patients demonstrated the lowest trigeminal sensitivity.

## 1 Introduction

Olfactory dysfunction (OD), which has a strong impact on daily life, affects up to approximately 20% of the general population, with 5% of them being anosmic [1, 2], and its prevalence increases in the elderly. OD may arise for a variety of reasons, such as aging, sinonasal diseases (SND), viral infections (PVOD), and trauma (PTOD). Various treatment options have been explored in the past, including medication, olfactory training, and surgery [2]. Whilst anosmia is highly debilitating, treatment options are currently limited and offer suboptimal results. Thus, new treatment approaches are needed [3], with the trigeminal system being an interesting option for further research due to the close interlink of the trigeminal and olfactory systems [4, 5, 6], as trigeminal stimulation can affect the perception of smell [6, 7, 8]. Additionally, it is known that numerous odors simultaneously activate both nerves [9], and the olfactory and trigeminal systems interact at central and peripheral levels of neural processing, indicating interdependence [10, 11].

Studies showed that trigeminal sensitivity declines with age [12, 13]. Similarly, trigeminal function is found to be decreased in patients with OD [14, 15]. These studies have been primarily interested in the synergy between the two systems and have tested the trigeminal system using chemosensory responses. However, the trigeminal system enables both chemosensory and somatosensory perception [16, 17]. Most of the existing literature is based on mechanical and chemical methods [18, 19, 20], with relatively few studies on electrical [21, 22] and thermal stimulation to date [23, 24]. As a result, the trigeminal somatosensory pathway has been much less studied. Trigeminal afferents transmit both chemosensory and somatosensory information to central structures, though the two systems function relatively independently [17]. Unlike chemical or thermal stimulation that activates specific receptors [25], electrical stimulation acts directly on the nerve fiber membranes, bypassing specific receptors. Further, patients with SND exhibit a higher electrical threshold compared to healthy individuals [22]. However, many aspects of the responsiveness of the nasal mucosa to electrical stimuli remain unclear.

The present study is the first study that aimed to: (i) compare the electrical thresholds between healthy individuals and patients with OD; (ii) investigate whether these thresholds vary with the etiology of OD (PIOD, PTOD, and SND) patients; and (iii) use electrical stimulation to characterize trigeminal somatosensory sensitivity as a function of age.

## 2 Materials and Methods

### 2.1 Participants

The multicentric study was conducted at three centers: Technische Universität Dresden (TUD, *n* = 40), Aristotle University of Thessaloniki (AUTH, *n* = 34), and the Center for Research in Neuroscience at Lyon (CRNL, *n* = 8) from March 2023 to July 2023. A total of 82 participants (age range: 18–83 years, mean 47.5 ± 16.5 years, 47 females, 35 males) were recruited for the study, consisting of 28 normosmic healthy individuals and 54 patients with OD of varying etiologies (SND: 10; PTOD: 15; PVOD: 29), of whom 32 were hyposmic and 22 anosmic (see Table 1).

**TABLE 1:** Demographics and results from participants.

Group	*N*	Gender (male/female)	Mean age ± SD (years)	Olfactory status (normosmics/hyposmics/anosmics)	Mean electrical threshold ± SD (mA)
Healthy	28	17/11	38.3 ± 16.4	28/0/0	0.73 ± 0.57
Patients	54	18/36	52.3 ± 14.8	0/21/33	1.83 ± 1.97
SND	10	7/3	50.0 ± 14.5	0/5/5	1.91 ± 1.54
PTOD	15	3/12	54.8 ± 8.3	0/7/8	2.96 ± 3.09
PVOD	29	8/21	51.8 ± 17.5	0/9/20	1.21 ± 0.86

Abbreviations: PTOD, post‐traumatic olfactory dysfunction; PVOD, post‐viral olfactory dysfunction; SND, sinonasal diseases.

Group of healthy individuals included: normosmics, as determined by the “Sniffin’ Sticks” battery test [26], with no history or clinical evidence of non‐inflammatory conditions affecting olfaction, such as PVOD, PTOD, or neurodegenerative diseases.

The inclusion criteria for patients were: adult patients with OD older ≥18 years and OD with a minimum duration of 6 months.

Exclusion criteria for the study were: acute upper respiratory infections, exacerbation of allergic rhinitis, neurodegenerative disorders, previous nasal surgery, systemic diseases associated with smell disorders like chronic renal failure, pacemaker use, pregnancy, and current or recent use of steroids.

SND group: All patients in this group had chronic rhinosinusitis with nasal polyps (CRSwNP).

PTOD group: Patients in this group had a history of traumatic brain injury (TBI). TBI severity was classified as Grade I (<30 min unconsciousness), Grade II (<1 h), and Grade III (>1 h) [27]. The study included seven patients with Grade I, four patients with Grade II, and four patients with Grade III TBI.

PVOD group: Patients were included in this group if they developed OD following symptoms of a viral upper respiratory infection (URI), in the absence of other identifiable causes.

### 2.2 Assessment of Olfactory Function

Olfactory function was evaluated using the extended version of the “Sniffin’ Sticks” battery test (Burghart Messtechnik GmbH, Holm, Germany) [26], assessing odor threshold (T), discrimination (D), and identification (I). According to this test, a score of 31 points or more indicated normosmia, between 16 and 31 points indicated hyposmia, and fewer than 16 points indicated anosmia [28]. Olfactory function was classified based on the terminology provided in the study Olfactory Nomenclature by Hernandez et al. [29].

### 2.3 Assessment of Trigeminal Function, Electrical Stimulation

**FIGURE 1:**
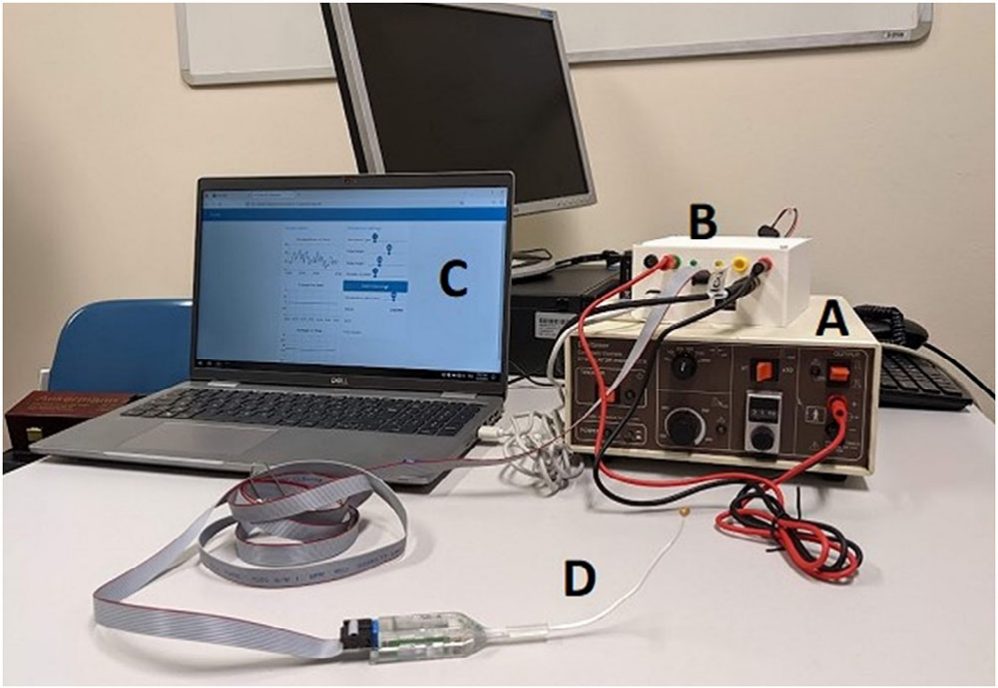
The electrical stimulation protocol. The Digitimer (A) produces the electrical stimulation. It is connected to a control box (B) that converts the output from the computer (C) to a signal that delivers stimulation through the electrode (D).

Intranasal trigeminal function was assessed by recording the sensitivity of the anterior septum to electrical stimuli. The trigeminal stimulator included a solid holder for electrical connection, a bendable rod (length 19 mm), and a head containing the stimulation device. The intranasal electrode was coupled to an electrical signal generator (Digitimer DS7A, Digitimer Ltd, UK) controlled by a computer via an electronic board with a customized microcontroller (see Figure 1). The electrical threshold testing procedure was standardized across institutions using identical devices, and an agreed‐upon protocol was prepared as described in the Procedure section.

### 2.4 Procedure

The study was conducted according to the Declaration of Helsinki and had been approved by the local ethics boards (TUD: BO‐EK‐74022022; AUTH: Prot. No.: 59718/2021; CRNL:IRB: IORG0009855). Each participant provided written informed consent following a comprehensive presentation of the study.

Following a detailed explanation of the study, the participants filled out their medical history, including questions on conditions with a possible effect on olfactory function. All experiments were conducted in air‐conditioned rooms with a temperature maintained between 21°C and 22°C. All participants were advised to avoid perfumes, eating/drinking, smoking, and chewing gum 1 h before the measurement.

**FIGURE 2:**
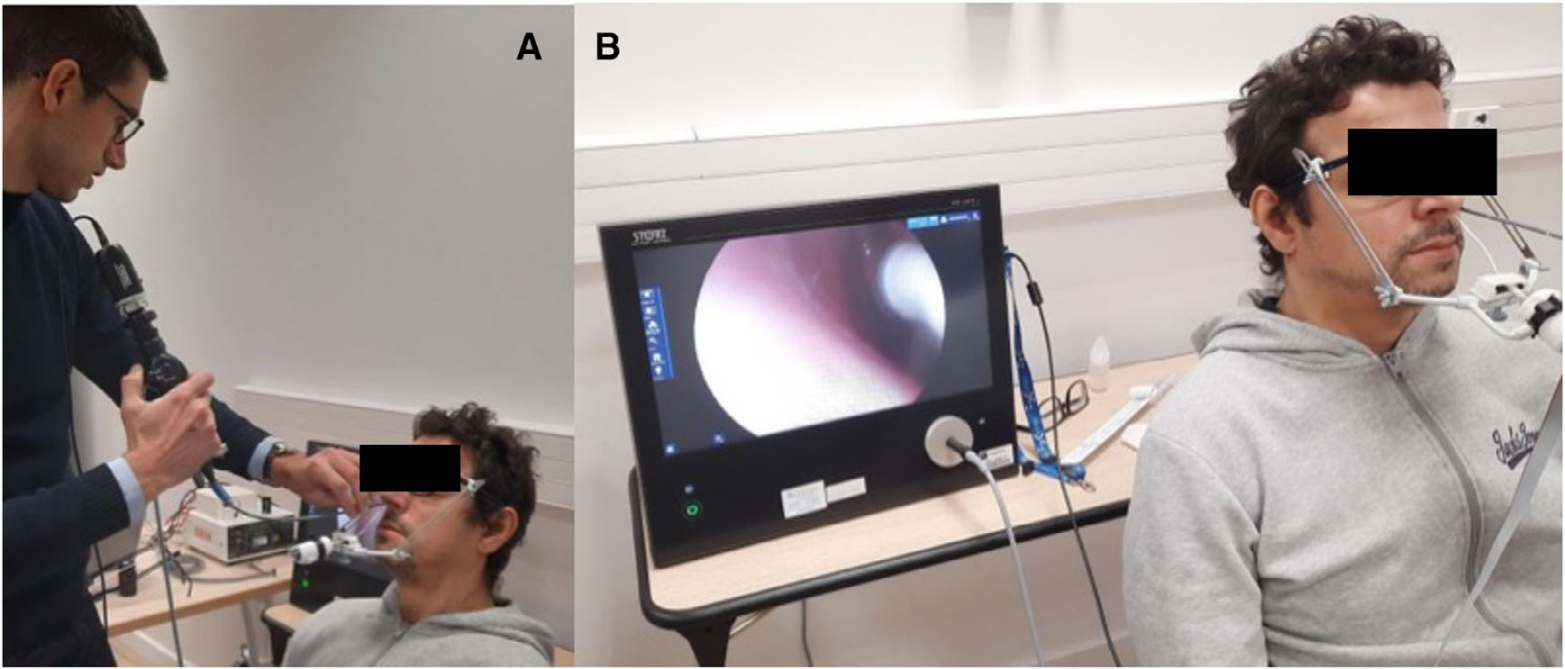
A) Placement of the electrode using an endoscope. (B) The electrode is stabilized by an external holder attached to a pair of lensless glasses.

The participant was comfortably seated in a chair with their head in a stable position and was asked to breathe quietly through their nose. The electrode was placed by an ENT specialist under nasal endoscopy (KG and IK at AUTH; SW at TUD; MF at CRNL). The tip of the electrode was placed in contact with the nasal mucosa at the anterior septum (1 cm from the vestibulum, 2 cm above the base of the nasal cavity). The stimulation was performed in the anterior septum, because this area is easily accessible and showed the highest chemosensory [19] and somatosensory sensitivity [21]. The electrode was held in place by an external holder attached to a pair of lensless glasses [30] (see Figure 2).

Threshold detection of the electrical stimulus was determined via a “staircase method” [31]. This procedure consisted of increasing the current intensity from 0.00 mA in steps of 0.10 mA until the participant detected the electrical stimulation twice in a row. The current intensity was then decreased in steps of 0.05 mA until the individual could no longer detect the stimulation. These turning points were established seven times (in 0.05 mA steps). The threshold was calculated by averaging the last four measurements. An inter‐stimulus interval of approximately 10 s between stimulations was used to limit possible habituation or over‐sensitization.

Participants were also asked to evaluate their subjective perception of pain, tickling, cold, warmth, and intensity using visual analog scales (VAS) ranging from 0 (no sensation) to 10 (maximum sensation) for two levels of electrical stimulation: (i) at the threshold level, and (ii) at double the threshold value. Additionally, they rated pleasantness on a VAS ranging from 0 (extremely unpleasant) to 10 (very pleasant).

### 2.5 Statistical Analysis

Statistical analyses were performed using IBM SPSS Statistics (Statistical Packages for the Social Sciences, version 28.0, SPSS Inc., Chicago, Illinois, USA) and the software Jamovi package. Descriptive statistics were obtained; continuous variables are expressed as means with standard deviation (SD), while categorical variables are presented as frequencies (percentages). We assessed data normality. As the skewness of all variables was less than 1.25, indicating no strong deviation from normality, parametric tests were applied [32].

A generalized linear mixed model (GLMM) was used to investigate the effects of age, TDI, and group of health status (healthy, PVOD, PTOD, or SND OD) on the electrical threshold at the anterior septum. The effects were calculated separately and together.

A *p*‐value of less than 0.05 was set as the level of statistical significance.

## 3 Results

### 3.1 Participants Characteristics: Age and Gender Versus Olfactory Status

Patients were significantly older (mean = 52.3 ± 14.8 years) than healthy individuals (mean = 38.3 ± 16.4 years; *t*(80) = −3.90, *p* < 0.001).

A pairwise comparison showed that healthy individuals were younger than each OD group (PVOD: *p* = 0.002, *M*_diff_ = 13.44, 95% CI [5.27, 21.60]; PTOD: *p* = 0.001, *M*_diff_ = 16.48, 95% CI [6.62, 26.34]; SND: *p* = 0.044, *M*_diff_ = 11.68, 95% CI [0.33, 23.03]). However, the OD groups did not differ in age.

Regarding gender, there were no significant age differences between genders (*p* = 0.239), but proportionally more women than men had OD (*χ* ^2^ = 5.65, *p* = 0.017).

### 3.2 Effect of Etiology of OD on Electrical Thresholds

**FIGURE 3:**
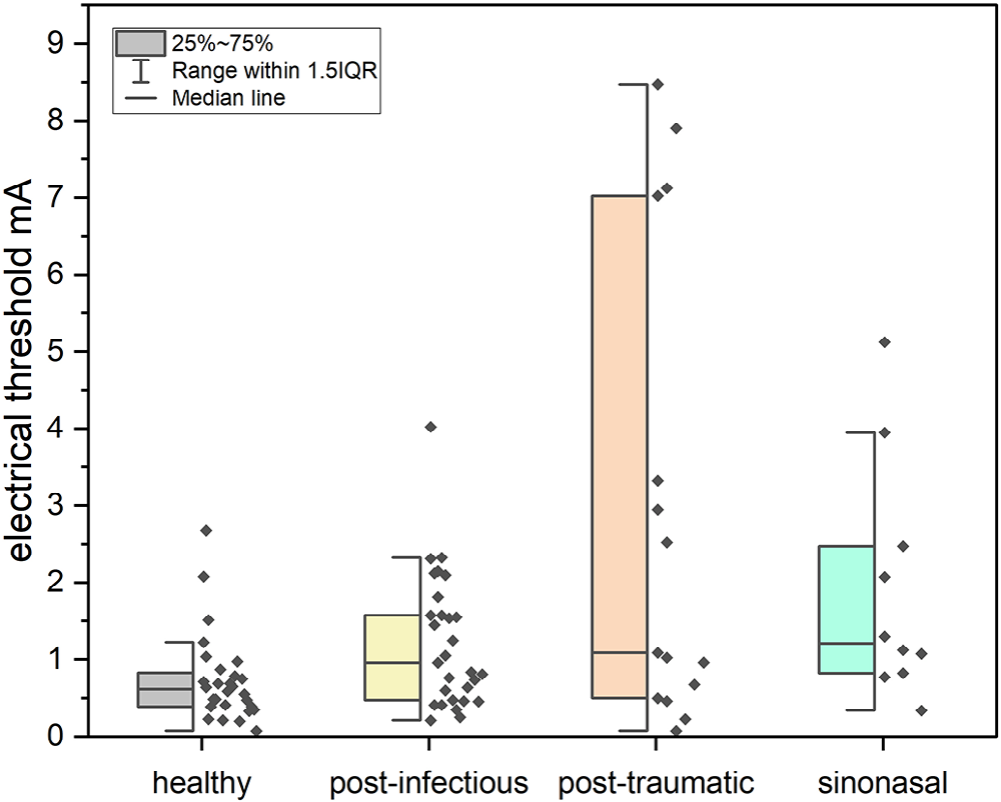
Electrical thresholds of patients with different etiologies of olfactory dysfunction (OD).

A significant effect of OD etiology on the electrical threshold was found (*F*(1,80) = 6.04, *p*< 0.001) (see Figure 3). Pairwise comparisons revealed a significant difference between healthy individuals and patients with PTOD (*t*(78) = 2.86, *p* = 0.033, 95% CI [0.12, 4.33]). Further, pairwise comparison showed no difference between healthy individuals and patients with PVOD (*t*(78) = 2.55, *p* = 0.064, 95% CI [0.018, ‐0.985]) or SND (*t*(78) = 2.47, *p* = 0.064, 95% CI [−2.40, 0.05]), nor between PVOD and PTOD patients (*t*(78) = 2.22, *p* = 0.089, 95% CI [−3.67, 0.18]).

When the comparison was restricted only to OD groups, a significant difference in the electrical threshold was observed (*F*(2,51) = 3.26, *p* = 0.046), with PTOD patients exhibiting the greatest variance (see Figure 3).

### 3.3 Effect of Olfactory Function Status on Electrical Thresholds

A significant effect of olfactory function, as measured by TDI scores, was found on the electrical threshold (*F*(1,72) = 12.34, *p* < 0.001), indicating that individuals with higher TDI scores had lower electrical thresholds.

**FIGURE 4:**
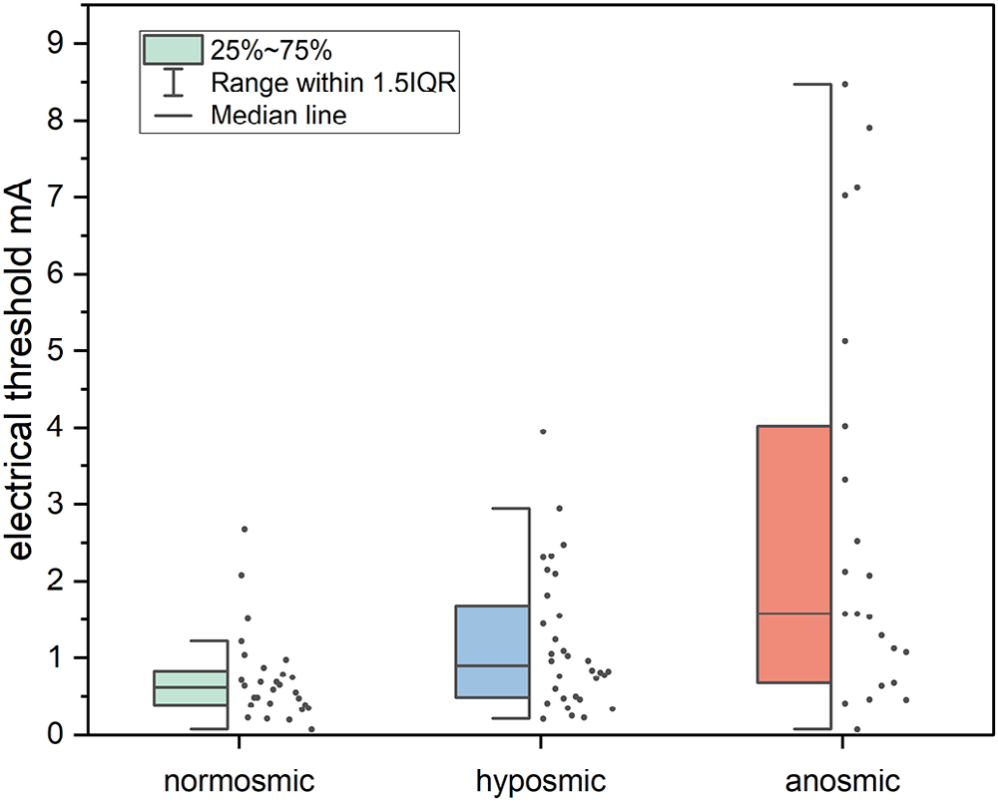
Electrical thresholds of healthy individuals, hyposmic, and anosmic patients.

A univariate analysis of variance showed a significant effect of electrical threshold across olfactory function status groups (normosmic, hyposmic, anosmic); *F*(2,79) = 11.72, *p* < 0.001. Pairwise comparisons revealed significant differences between normosmic individuals and anosmic patients (*M*_diff_ = 2.03, *p* < 0.001, 95% CI [0.97, 3.09]), as well as between hyposmic and anosmic patients (*M*_diff_ = 1.57, *p* = 0.001, 95% CI [0.54, 2.60]); however, hyposmic patients did not differ from normosmic individuals (*M*_diff_ = 0.45, *p* = 0.075, 95% CI [−0.51, 1.42]) (see Figure 4).

### 3.4 Effect of Age on Electrical Thresholds

Across all participants (healthy individuals and patients), age had a significant effect on the electrical threshold (*F*(1,80) = 6.67, *p* = 0.012), with older participants exhibiting a higher electrical threshold.

For the 28 healthy individuals, there was a positive correlation between age and the detection threshold of stimulation (Pearson's *r* = 0.245, *p* = 0.02, 95% CI [0.03, 0.44]). For all patients, regardless of OD etiology, there was no correlation between age and electrical threshold (Pearson's *r* = 0.11, *p* = 0.43). Similarly, no correlation was found between age and electrical threshold for the different etiologies: PVOD (Pearson's *r* = −0.051, *p* = 0.793), PTOD (Pearson's *r* = 0.18, *p* = 0.52), and SND (Pearson's *r* = 0.38, *p* = 0.28).

When the effects of olfactory function (TDI), age, and etiology of OD on the electrical threshold were considered together, only the significant effect of olfactory function remained (*F*(1,68) = 6.45, *p* = 0.013). However, there was a significant interaction between TDI and the etiology of OD (*F*(3,61) = 4.31, *p* = 0.008).

### 3.5 Perceptual Ratings

**FIGURE 5:**
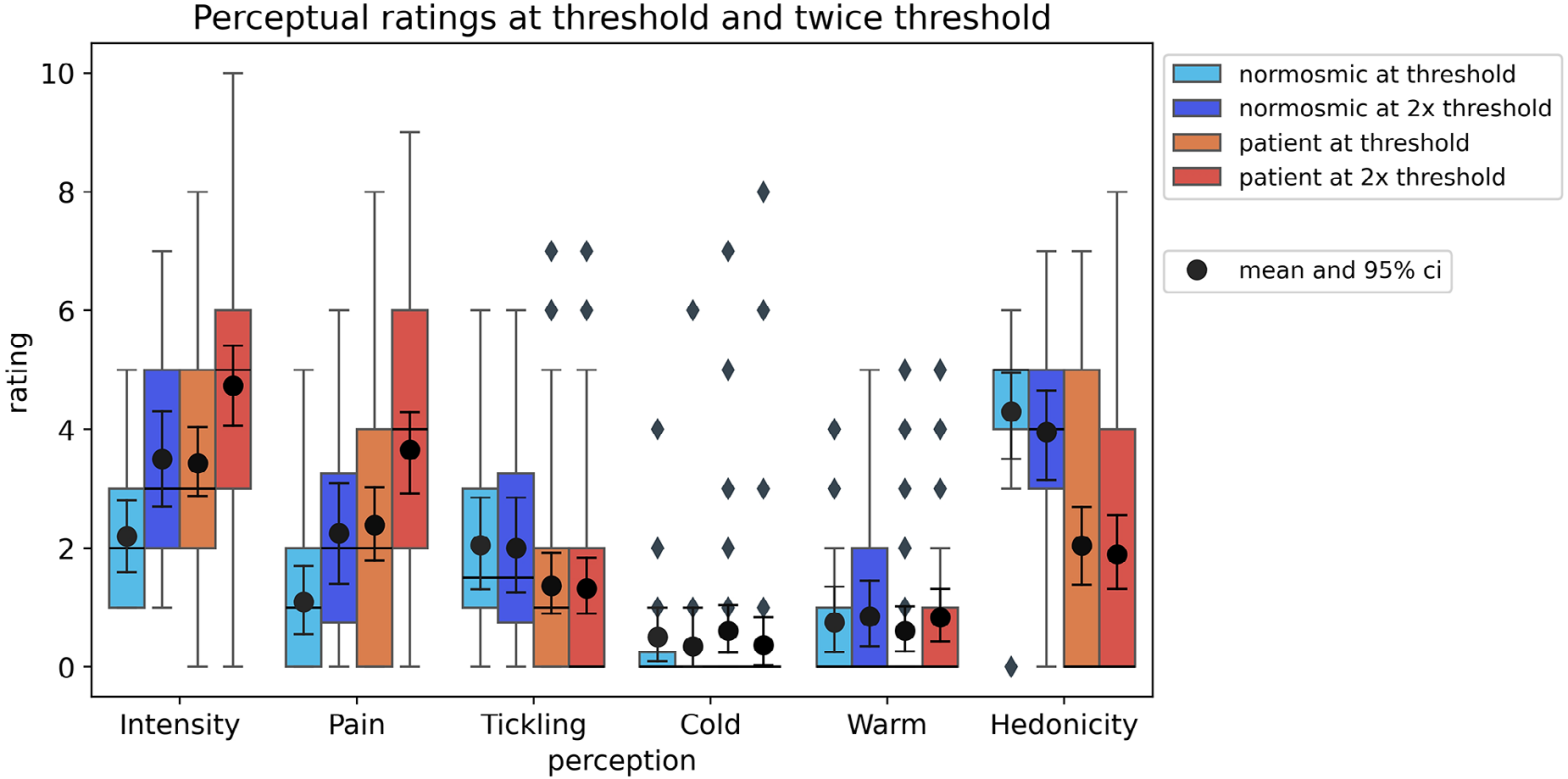
Perceptual ratings at threshold and twice‐threshold currents by olfactory status were assessed using a visual analog scale. Ratings ranged from 0 (nothing) to 10 (very intense, very painful, very tickling, very cold, or very warm). For pleasantness, ratings ranged from 0 (unpleasant) to 10 (pleasant), with 5 representing neutral.

Both healthy individuals and patients evaluated the electrical stimulations as moderately intense and mildly painful, with intensity and pain increasing as the stimulation current increased (see Figure 5). There was a slight perception of tickling, but no sensation of cold or warmth. No significant differences were found in ratings of cold, warmth, tickling, or hedonicity as the current increased.

However, at both threshold and 2×‐threshold currents, patients perceived higher intensity, more pain, and lower pleasantness than healthy individuals (*p* < 0.05) (see Figure 5). No significant differences were found between hyposmic and anosmic individuals.

## 4 Discussion

In this large multicentric study, somatosensory stimulation of the intranasal trigeminal system via electrical stimulation of patients with OD and healthy participants resulted in the following major findings.

1. The somatosensory trigeminal function seems to be preserved in the majority of patients. Specifically, the sensitivity of PVOD and SND patients did not differ significantly from that of healthy individuals. An exception to this was the PTOD patients, who exhibited the lowest trigeminal sensitivity compared to healthy individuals.
2. Anosmic patients had worse trigeminal function compared to hyposmic and normosmic individuals, while hyposmic individuals did not differ significantly from normosmic individuals. Among age, etiology of OD, and olfactory function status, the latter appears to be the most significant factor affecting trigeminal somatosensory sensitivity.
3. Age significantly impacted trigeminal somatosensory sensitivity, with the effect observed when healthy individuals and patients were combined, but only evident in healthy individuals when considered separately.
4. Both healthy participants and patients similarly perceived electrical stimulations, describing them as moderately intense and mildly painful. Additionally, they reported that stimulations became more intense and painful as the stimulation current increased.

The present study, except for PTOD patients, shows a relatively well‐preserved somatosensory trigeminal system in patients with PVOD and SND. This is partially in contrast with trigeminal chemosensitivity, which follows the pattern of olfactory impairment [33]. This can be attributed to the fact that both olfactory and trigeminal stimuli activate similar brain areas, such as the ventral insula, middle frontal gyrus, dorsolateral orbitofrontal cortex, superior temporal gyrus, and caudate nucleus [34]. In contrast, electrical stimulation evokes activation patterns that partially overlap with those induced by chemical stimuli, mainly activating the secondary somatosensory cortex, the insula, and the thalamus, [50]. Another hypothesis regarding the different reactions of trigeminal somatosensory function to OD could be peripheral changes due to a specifically reduced susceptibility of trigeminal chemoreceptors in OD patients. Both chemosensory and electrical stimuli activate the same subset of A and C fibers [18, 35]. However, at low intensities, as in our study, they primarily activate A fibers, which contradicts the theory that different fiber activation explains the somatosensory system's different behavior in OD [18].

The present study showed that PTOD patients had lower somatosensory sensitivity on average. This is not in agreement with Frasnelli et al., who found no difference between healthy subjects and patients with PTOD and PVOD using electrical cutaneous stimulation of the cutaneous maxillary branch of the trigeminal nerve [18]. However, in the same study, PTOD patients had lower chemosensitivity than healthy subjects. In addition, our data seem to be inconsistent with the results of Poletti et al., which showed decreased trigeminal sensitivity for SND compared to healthy individuals [22]. However, their results show that the anterior part of the nose thresholds in healthy subjects and SND patients are quite similar (being closer on the lateral wall than on the septum, where the mean percentage difference was about 33%). Upregulated immune defense mechanisms, such as altered nerve growth factor release or damage to nerve endings by neuroinflammation, may contribute to trigeminal dysfunction in SND [36]. Specifically, for PTOD patients, there is evidence that chemosensory trigeminal sensitivity was more severely impaired than in other etiologies [14, 37, 38]. This may explain why somatosensory sensitivity shows a similar decline as the negative impact of trauma in central structures of the trigeminal system can cause more severe damage than the peripheral, mainly seen in PVOD and SND.

In the Frasnelli et al. study on chemosensory and somatosensory sensitivity, the authors found that the trigeminal response of patients varies depending on both etiology and degree of olfactory dysfunction [18]. Furthermore, Migneault‐Bouchard et al. demonstrated a relationship between age, the cause of OD, and chemosensory function in general (smell, taste, trigeminal sensitivity) [33]. In the same study, when trigeminal chemosensory sensitivity was analyzed separately, age had a greater effect than the cause of OD, which aligns with our findings. However, this study showed that PTOD patients exhibited the most severe trigeminal dysfunction, and the PVOD had the best trigeminal function, followed by SND patients, similar to our results. This may be attributed not only to damage in olfactory areas such as the orbitofrontal cortex but also to subclinical injury of trigeminal afferents [18].

Our findings regarding the effects of age on somatosensory trigeminal sensitivity align with the findings for chemosensory responses, suggesting an age‐related decline [12, 33]. Chemosensitivity and aging were objectively assessed using event‐related potentials (ERPs) in previous studies, which showed a decline in both healthy individuals and patients [37, 39]. Similar results in chemosensitivity, with an age‐related decrease in normosmic individuals, have been published, especially using psychophysical testing [40, 41]. For example, in the study by Feit et al., over one‐third of aging adults were found to have impaired trigeminal sensitivity on the 60% concentration eucalyptol lateralization task [42]. These similarities to the age‐related changes observed in olfactory function and trigeminal chemosensitivity can be expected, as both systems, while independent neurologically, interact at different levels—from the periphery to central structures—to produce the sensation of smell [5]. However, there is a lack of evidence on somatosensory stimulation of the nasal cavity. In a pain perception meta‐analysis, an aging‐related decline in pain sensitivity has been observed, mainly for thermal but not for electrical stimuli. However, in the same study, a much more relevant finding was the accumulation of positive findings for an age‐related increase in pain threshold when stimulation was applied to areas of the head, including soft tissues and teeth. The authors stated that this finding suggests a stronger age effect on the trigeminal system than on the spinal nociceptive system [43]. Aging also affects tactile detection thresholds, as seen in a study assessing trigeminal somatosensory sensitivity on the skin overlying the mental foramen using mechanical stimuli [44].

The intranasal trigeminal nerve can be activated by mechanical, thermal, or chemical stimulation [23]. Several methods have been described to assess intranasal trigeminal sensitivity, including psychophysical and electrophysiological approaches [25, 45]. Each method evaluates a different aspect of trigeminal nerve function [23], and the results often depend on the anatomical site where the assessment is performed [45, 46, 47]. Konstantinidis et al. found a positive correlation between nasal cavity size and trigeminal event‐related potentials in response to suprathreshold CO_2_ and menthol stimuli. They suggested that at least at suprathreshold levels, nasal anatomy plays a role in determining interindividual differences in sensitivity to trigeminal stimuli [47]. The anterior and superior parts of the nasal cavity are generally more sensitive to chemical stimulation than the posterior regions [19, 46]. Frasnelli et al. showed that CO_2_ stimulation elicits more intense responses in the anterior part of the nasal cavity compared to the posterior [46]. Similarly, electrophysiological studies support that the anterior nasal septum is more responsive to stimulation [19, 20, 21]. Mechanical stimuli assessed via airflow have been found to evoke stronger responses in the nasal vestibule [48]. For thermal stimulation, thermal thresholds were found to differ between sites, with a trend toward higher sensitivity at the anterior septum [23]. In another study, the inferior turbinate appeared to correlate more strongly with the septum than the mouth and face [24]. Finally, electrical stimulation showed higher sensitivity in the anterior part of healthy noses compared to other regions [21].

In general, healthy subjects and patients seem to perceive the given list of sensations in a similar pattern, giving lower ratings to warm and cold, followed by tickling, and higher ratings to pain sensations. Patients rated the stimuli significantly more intense, more painful, and less pleasant than healthy individuals. This reveals a relative discrepancy between the somatosensory threshold and the corresponding subjective ratings. A possible explanation could be related to contextual differences between the two groups, with patients typically receiving significantly less information through their nasal senses compared to healthy individuals. In the present experimental context, however, they were asked to pay attention to their nasal sensitivity and then, experiencing sensations that are possibly disregarded in daily life, these sensations were rated relatively highly. Another explanation might relate to possible compensatory mechanisms in the trigeminal system as a consequence of olfactory loss, similar to what has been reported in the tactile system in visual impairment [49]. Further, there is evidence suggesting that electrical trigeminal stimulation activates different brain structures compared to chemosensory or mechanical stimulation, which affects the evoked sensation [50].

OD presents limited therapeutic options, namely olfactory training, medications, and surgical approaches based on the underlying cause [2, 51]. However, results are frequently unsatisfactory. For example, the recovery rate for persistent PVOD has been reported at 32%–36% over 15 months. In the context of PTOD, outcomes are even less promising [52, 53, 54], with an improvement of only about 10%–25% within roughly the same timeframe [52, 53]. In patients with OD who do not respond to any treatment, the somatosensory trigeminal system seems to be a promising pathway for stimulation in the absence (or deficiency) of the olfactory system and preservation of the trigeminal system. A previous report showed that electrical stimulation of the trigeminal nerve via trigeminal nerve stimulation or transcranial direct current stimulation improved sensitivity to guaiacol in healthy individuals [6]. Olfactory implants could serve in the future as a therapeutic option, such as cochlear or retinal implants. Similarly, implants that produce electrical stimulation of the intranasal trigeminal system could be designed as an alternative pathway to stimulate an impaired olfactory system, provided the trigeminal system remains relatively intact. Identifying suitable candidates requires further research; based on current results, young, hyposmic, and non‐PTOD patients appear the most promising.

One limitation of our study is the heterogeneity of the study population. Specifically, the number of participants in certain subgroups, such as those with SND and PTOD, was relatively small, which may limit the generalizability of subgroup‐specific findings. Additionally, there was an age discrepancy between patient groups and healthy controls, with patients being generally older. To account for this potential confounder, age was included as a covariate in the generalized linear mixed model (GLMM). Nevertheless, residual confounding cannot be completely ruled out. Future studies with larger, more balanced, and age‐matched cohorts are needed to validate and extend these findings.

## 5 Conclusion

The present study suggests that the intranasal somatosensory trigeminal system remains preserved in most patients with OD, particularly in those with hyposmia. Among the different OD etiologies, PTOD patients exhibited the lowest trigeminal sensitivity. Additionally, trigeminal somatosensory sensitivity generally declined with age. The functionality of the somatosensory trigeminal system in OD seems to be a promising platform for future therapeutic options in patients with OD.

## Author Contributions

Conceptualization: Moustafa Bensafi, Thomas Hummel, Iordanis Konstantinidis. Data curation: Konstantinos Garefis, Evangelia Tsakiropoulou, Iordanis Konstantinidis, Susanne Weise, Pauline Hanslik, Coralie Mignot, Halina B. Stanley, Maxime Fieux. Formal analysis: Halina B. Stanley, Moustafa Bensafi, Konstantinos Garefis, Susanne Weise, Thomas Hummel, Iordanis Konstantinidis. Investigation: Konstantinos Garefis, Evangelia Tsakiropoulou, Susanne Weise, Pauline Hanslik, Coralie Mignot, Halina B. Stanley, Maxime Fieux, Camille Ferdenzi, Clementine Lipp, Arnaud Bertsch, Moustafa Bensafi, Iordanis Konstantinidis, Thomas Hummel. Methodology: Konstantinos Garefis, Evangelia Tsakiropoulou, Susanne Weise, Halina B. Stanley, Maxime Fieux, Clementine Lipp, Arnaud Bertsch, Moustafa Bensafi, Iordanis Konstantinidis, Thomas Hummel. Project administration: Moustafa Bensafi, Thomas Hummel, Iordanis Konstantinidis. Supervision: Moustafa Bensafi, Thomas Hummel, Iordanis Konstantinidis. Writing—original draft: Konstantinos Garefis, Iordanis Konstantinidis, Halina B. Stanley, Susanne Weise. Writing—review and editing: Konstantinos Garefis, Evangelia Tsakiropoulou, Susanne Weise, Pauline Hanslik, Coralie Mignot, Halina B. Stanley, Maxime Fieux, Camille Ferdenzi, Clementine Lipp, Arnaud Bertsch, Moustafa Bensafi, Iordanis Konstantinidis, Thomas Hummel.

## Conflicts of Interest

The authors declare no conflicts of interest.
